# Visuo-tactile interactions in the congenitally deaf: a behavioral and event-related potential study

**DOI:** 10.3389/fnint.2014.00098

**Published:** 2015-01-21

**Authors:** Nadine Hauthal, Stefan Debener, Stefan Rach, Pascale Sandmann, Jeremy D. Thorne

**Affiliations:** ^1^Neuropsychology Lab, Department of Psychology, Cluster of Excellence “Hearing4all,” European Medical School, University of OldenburgOldenburg, Germany; ^2^Research Center Neurosensory Science, University of OldenburgOldenburg, Germany; ^3^Experimental Psychology Lab, Department of Psychology, European Medical School, University of OldenburgOldenburg, Germany; ^4^Department of Epidemiological Methods and Etiologic Research, Leibniz Institute for Prevention Research and Epidemiology – BIPSBremen, Germany; ^5^Department of Neurology, Cluster of Excellence “Hearing4all,” Hannover Medical SchoolHannover, Germany

**Keywords:** deafness, cross-modal plasticity, multisensory processing, race model, redundant signals effect

## Abstract

Auditory deprivation is known to be accompanied by alterations in visual processing. Yet not much is known about tactile processing and the interplay of the intact sensory modalities in the deaf. We presented visual, tactile, and visuo-tactile stimuli to congenitally deaf and hearing individuals in a speeded detection task. Analyses of multisensory responses showed a redundant signals effect that was attributable to a coactivation mechanism in both groups, although the redundancy gain was less in the deaf. In line with these behavioral results, on a neural level, there were multisensory interactions in both groups that were again weaker in the deaf. In hearing but not deaf participants, somatosensory event-related potential N200 latencies were modulated by simultaneous visual stimulation. A comparison of unisensory responses between groups revealed larger N200 amplitudes for visual and shorter N200 latencies for tactile stimuli in the deaf. Furthermore, P300 amplitudes were also larger in the deaf. This group difference was significant for tactile and approached significance for visual targets. The differences in visual and tactile processing between deaf and hearing participants, however, were not reflected in behavior. Both the behavioral and electroencephalography (EEG) results suggest more pronounced multisensory interaction in hearing than in deaf individuals. Visuo-tactile enhancements could not be explained by perceptual deficiency, but could be partly attributable to inverse effectiveness.

## Introduction

For many decades, the sensory systems were studied in isolation to learn about the function of the visual, the auditory, and the tactile systems, amongst others. However, sensory systems do not function independently but rather influence each other (for reviews, see e.g., Ghazanfar and Schroeder, 2006; Driver and Noesselt, 2008). An interesting question is whether multisensory processing, that is, the processing of one input in the presence of another, can be affected by sensory deprivation. Deafness, for example, provides an opportunity to investigate the effects of altered sensory experience, namely auditory deprivation, on the remaining intact sensory systems. Deafness is mostly defined in terms of a deficit view. However, deafness is more than simply “not hearing.” Many deaf do not see themselves as impaired, but as members of a community with its own language, its own identity, and its own culture that needs to be preserved (see e.g., Paddy, 2003). In this spirit, the present study is not focusing on what is not working, but focusing on what might simply be different with deafness. How do the intact visual and tactile systems change due to altered sensory experiences as with deafness? More precisely, do the deaf see or feel better than hearing individuals? Are there differences in integrating visual and tactile information? Moreover, knowledge about the functioning of a neural system when one sensory input is missing also helps the understanding of the functioning of the intact neural system. The question of the extent to which the contribution of all sensory systems during maturation is necessary to develop the ability to integrate information from different sensory systems could be answered by looking at individuals who are deprived of one sensory input, such as the deaf.

In deaf research, the bulk of existing studies has examined consequences for the *visual* system (for a review, see Pavani and Bottari, 2011). Previous studies have reported enhanced performance of deaf compared to hearing participants in detecting visual stimuli (e.g., Loke and Song, 1991; Reynolds, 1993; Bottari et al., 2010; Heimler and Pavani, 2014). Electrophysiological findings similarly point to a difference in visual processing between deaf and hearing individuals (e.g., Neville and Lawson, 1987; Armstrong et al., 2002; Bottari et al., 2011, 2014; Hauthal et al., 2014). However, not much is known about tactile processing. Levänen and Hamdorf (2001) showed better *tactile* change detection in deaf than in hearing participants, but found no difference between the two groups in tactile frequency discrimination. Furthermore, tactile detection thresholds were reported to be either comparable in deaf and hearing individuals (Moallem et al., 2010) or elevated in the deaf (Frenzel et al., 2012). Tactile detection, measured by response times, was comparable in deaf and hearing participants (Heimler and Pavani, 2014). Currently, there is no study comparing tactile processing between deaf and hearing adults using EEG (see Charroo-Ruiz et al., 2012, 2013 for studies in deaf children).

To date, the only study that has investigated visuo-tactile processing in the deaf is that of Karns et al. (2012) who used an adapted version of the Shams' illusion (Shams et al., 2005). Participants had to detect double flashes within a series of single flashes or double touches within a series of single touches. When in the so called illusory trials the single flashes were accompanied by two touches they were perceived as double touches by the deaf. This was, however, not the case for hearing participants, contradicting previous studies in hearing individuals (e.g., Violentyev et al., 2005; Lange et al., 2011), which might have been related to the difference in the response mode compared to the classical version of the paradigm in which a response about the number of perceived touches is required. Since the deaf, on the other hand, did show the touch-induced double flash illusion, it was concluded that visuo-tactile interaction was found in the deaf only. Furthermore, in the deaf, the size of this multisensory illusion was positively related to somatosensory and bisensory activation in the auditory cortex, suggesting cross-modal reorganization, that is, the recruitment of deprived auditory areas by the intact visual and tactile systems. In contrast, research on the blind has revealed evidence for reduced multisensory interaction with visual deprivation. These studies have reported audio-tactile illusions to be not existent or less pronounced in blind compared to sighted participants (Hötting and Röder, 2004; Champoux et al., 2011; Occelli et al., 2012; but see Collignon et al., 2009, uncrossed hand posture). In general, one should be careful in directly comparing findings of studies with deaf and with blind participants. Although both deafness and blindness involve sensory deprivation, the differences of the visual and the auditory system in spatial and temporal characteristics make a direct comparison rather difficult (see e.g., Thorne and Debener, 2014, for a discussion on functional differences). Nevertheless a review of the literature on visual deprivation may be informative in understanding changes that follow auditory deprivation. Results regarding multisensory processing in deaf and blind individuals diverge, with visuo-tactile interactions being reported as more pronounced in deaf than hearing individuals and audio-tactile interactions as less pronounced in congenitally blind than sighted individuals. In the following, we refer to *multisensory interaction* as the interplay between inputs from different sensory systems. That is, if there is multisensory interaction in the present study, the visual and the tactile modalities will have altered each other's processing. *Multisensory integration* is referred to as the process by which unisensory inputs are combined to form a new integrated product (see Stein et al., 2010).

The present study aimed to better understand the effects of auditory deprivation on the remaining sensory systems. Congenitally deaf and hearing participants had to detect unisensory visual (V) and unisensory tactile (T) stimuli, as well as a combination of both (VT, i.e., bisensory redundant signals) while reaction times (RTs) and EEG were recorded. Firstly, we contrasted behavioral and electrophysiological responses to either visual or tactile stimulation between deaf and hearing participants. Secondly, we examined whether both groups showed a redundant signals effect, reflected by faster RTs, on average, when visual and tactile stimuli were presented together, compared to presenting each alone (e.g., Hecht et al., 2008; Girard et al., 2011). It was also studied whether any redundancy gain was similar in the two groups. Further, the race model inequality (Miller, 1982) was used to test whether any redundancy gain observed in deaf and hearing individuals could be attributable to a coactivation mechanism (see Section Behavioral Data for more details). Visuo-tactile interaction in event-related potentials (ERPs) was assessed by applying the additive model, based on the principle of superposition of electrical fields (see also Giard and Peronnet, 1999; Foxe et al., 2000; Molholm et al., 2002; Mishra et al., 2010; Senkowski et al., 2011). To this end, each unisensory response was used separately as a baseline comparison for the multisensory response, to assess whether one sensory system influences the other to a greater or lesser extent in deaf compared to hearing participants (see Section Analysis of ERPs for more details).

## Materials and methods

### Participants

Seventeen deaf individuals with differing hearing loss onset (0–6 years) took part in the experiment. The criterion for inclusion was a binaural hearing loss of at least 90 dB hearing level for the better ear as measured by pure tone audiometry (average at 0.5, 1, and 2 kHz). To improve homogeneity and to allow comparisons with other studies of deaf individuals that have used visual and tactile stimuli (Karns et al., 2012; Heimler and Pavani, 2014) all analyses reported here were performed on the congenitally deaf participants only. Thus, data from six participants who had become deaf after birth were excluded. Moreover, the behavioral responses of one congenitally deaf participant were more than four standard deviations above the mean (*M* = 359 ms, *SD* = 58 ms), so she was excluded from the analyses. Thus, the experiment included 10 congenitally deaf participants (mean age: 43 years, *SD* = 7 years, 7 male). The hearing impairments were of different etiologies. All deaf participants were fluent users of German Sign Language. More details for the deaf participants are listed in Table 1.

**Table 1:** **Details about congenitally deaf participants**.

**Subject**	**Sex**	**Age (years)**	**Cause of deafness**	**Age at Acquisition of DGS^a^ (years)**
1	M	36	Genetic	6–8
2	M	57	Unknown	18
3	F	42	Maternal rubella	3–4
4	F	43	Unknown	6–8
5	M	37	Genetic	0^b^
6	M	53	Unknown	6–8
7	M	38	Maternal rubella	6–8
8	M	38	Maternal rubella	6–8
9	M	45	Unknown	6–8
10	F	42	Maternal rubella	6–8

*F, female; M, male*.

a *DGS = German Sign Language*.

b *This participant learned sign language from his deaf parents. The remaining participants were communicating orally and via lip reading before they acquired DGS. Until then they were only using some signs*.

Seventeen hearing individuals also took part in the study. These control participants were required to have an average auditory threshold no worse than 20 dB hearing level (pure-tone average at 0.5, 1, and 2 kHz). Only the 10 hearing controls matched to the congenitally deaf participants were included in the analysis (mean age: 43 years, *SD* = 9 years, 7 male). None of the hearing participants had any knowledge of sign language.

All participants were right-handed and had normal or corrected-to-normal vision as tested using Landolt rings. None of the participants played any action video games (as enhancements in visual attention for habitual action video game players have been reported; for a review, see Dye et al., 2009). All participants gave informed consent and were paid for their participation. The study was carried out in accordance with the Declaration of Helsinki principles and approved by the ethics committee of the University of Oldenburg.

### Stimuli and procedure

Participants were comfortably seated in a quiet and dimly-lit room at a viewing distance of 120 cm. Responses were indicated through a device placed on the floor. Participants were barefoot and the ball of their right foot rested on a protruded button. A response was indicated by lifting the foot. Stimulus presentation was controlled by Presentation 14.8 software (Neurobehavioral Systems) on a personal computer, using a 61 cm monitor for visual stimulation (1920 × 1080 × 32 bit, 120 Hz refresh rate). Tactile stimuli were presented through two piezoelectric tactile stimulators (QuaeroSys Medical Devices) with one stimulator taped to each index finger of the participant. Each tactile stimulator consisted of a 2 × 4 matrix of pins. Stimulation was implemented by the eight pins extending and retracting simultaneously at a frequency of 55 Hz. The faint operating noise of the tactile device was attenuated by putting each participant's hand and the attached tactile stimulator in a mitten. Both deaf and hearing participants wore closed cup hearing protectors. Additionally, hearing participants wore foam ear plugs. None of the hearing participants reported any perceived noise by the tactile device.

Stimuli were presented in a unisensory visual, a unisensory tactile, and a bisensory visuo-tactile condition. A weak stimulation was chosen, because Senkowski et al. (2011) have shown that multisensory integration is more likely to occur in response to stimuli with low intensity. The unisensory visual stimulus was a white disk (diameter = 0.6° of visual angle) on the left or right of a central fixation cross at a peri-foveal eccentricity of 4.0° visual angle. Stimuli were presented on a gray background. A weak vibration to the left or right index finger served as unisensory tactile stimulus. The intensity of the tactile stimuli was the same for all participants. Bisensory stimulation was implemented via the simultaneous presentation of the visual and the tactile stimulus. In the bisensory condition, visual and tactile stimuli were always presented on the same side. Moreover, there was a condition with no stimulus being presented (for a rationale, see Section Analysis of ERPs).

Each trial started with a fixation cross presented in the center of the screen. After a jittered interval of 800–1300 ms, either a unisensory visual (V), a unisensory tactile (T), a bisensory visuo-tactile (VT), or no stimulus (nostim) appeared. The duration of the visual stimulus was 50 ms. The tactile stimulus lasted for 45 ms. In the bisensory condition the visual stimulus onset preceded the tactile stimulus onset by 4 ms as technical constraints prevented exact simultaneity. The response window was 1300 ms from stimulus onset. The fixation cross remained on the screen for the whole trial until the response window had finished. A blank screen then appeared for 800 ms before a new trial started.

Participants were instructed to respond as fast as possible to each stimulus irrespective of the sensory modality, and not to respond in cases of no stimulation. Participants were told to keep their fixation during the whole experiment. Hearing participants received written instructions. Deaf participants received instructions in written and sign language. In sum there were 480 trials presented in randomized order, with a break after each 120 trials. There were 120 trials per condition (V, T, VT, nostim). Feedback was given for absent responses. Participants completed 24 practice trials containing all four conditions. Participants who still did not feel comfortable with the task had the chance to do another 24 practice trials.

### Electroencephalography recording

The EEG data were collected from 96 Ag/AgCl electrodes using a BrainAmp system (BrainProducts, Gilching, Germany) and a customized, infracerebral electrode cap with an equidistant electrode layout (Easycap, Herrsching, Germany). Two of the 96 electrodes were placed below the eyes. Data were recorded with a sampling rate of 1000 Hz (analog time constant 10 s, low-pass 250 Hz), using a nose-tip reference and a midline site slightly posterior to Fz as ground. Electrode impedances were maintained below 20 kΩ before data acquisition.

### Data analysis

#### Behavioral data

Trials with RTs less than 100 ms were excluded from the analysis (0.2%) as well as trials for which no response was given (0.8%). Additionally, trials with a RT higher than three standard deviations above the individual mean were removed (1.5%). Mean RTs were calculated for each participant in each condition. Performance of deaf and hearing participants was compared in the visual (V), tactile (T), and visuo-tactile (VT) stimulus conditions. Therefore, mean RTs were entered into a 3 × 2 mixed ANOVA with Stimulus condition (V, T, VT) as within-subjects factor and Group (deaf, hearing) as between-subjects factor. In case of a significant interaction between the two factors, *post-hoc* two-tailed *t*-tests were conducted.

To explain the observed RT gain to redundant compared to unisensory signals, Raab (1962) proposed a *race model* for simple RTs that predicts faster RTs on redundant signals as a consequence of statistical facilitation: assuming that each of the redundant signals is processed in a separate channel and that the RT is solely determined by the process that finishes first, he could show that, in the absence of any coactivation mechanism, the time to respond to the first of several redundant signals is faster, on average, than the RT to any single signal. Miller (1982) introduced the race model inequality (RMI):

$$\text{P}({\text{RT}}_{\text{VT}}\leq \text{t})\;\leq \text{ P}({\text{RT}}_{\text{V}}\leq \text{t})\;+\text{ P}({\text{RT}}_{\text{T}}\leq \text{t}),\text{ for all t}\geq \text{0},$$

where P (RT_x_ ≤ t) represents the probability of an RT in condition x being below an arbitrary value t. This inequality stipulates that the RT distribution function for visuo-tactile stimuli can never be larger than the sum of the RT distribution functions of the visual and the tactile stimuli, unless some kind of coactivation mechanism is involved. Thus, it puts an upper limit to the RT gain that can be explained by statistical facilitation (i.e., probability summation) alone. Any violation against the race model inequality therefore indicates that the stimuli are not processed in separate channels, that is, there is evidence of an underlying coactivation mechanism. In multisensory research the race model inequality has become a standard tool to identify evidence of interaction between sensory systems in RT data. To analyze the race model inequality, we used the RMITest software (Ulrich et al., 2007). For each participant and each stimulus condition (V, T, VT) as well as for the bounding sum (V + T), the cumulative density function (CDF) of the RT distributions was estimated. RTs for each stimulus condition (V, T, VT) and for the bounding sum (V + T) were rank ordered to calculate percentile values (Ratcliff, 1979). We used a bin width of 10 per cent (cf. Sperdin et al., 2009; Girard et al., 2011). In each group, the redundant signals condition (VT) and the bounding sum (V + T) were then compared for the five fastest deciles (10-percentile bins: 0–50%) using one-tailed within-subjects *t*-tests with Bonferroni correction to adjust for multiple comparisons. If the RTs for redundant signals (VT) were significantly faster than those for the bounding sum (V + T) at any decile bin, the race model was violated and could not account for the facilitation in the redundant signals condition.

CDFs of the visuo-tactile condition (VT) and the bounding sum (V + T) were then entered into a 2 × 5 × 2 mixed ANOVA with Stimulus type (VT, V + T) and Decile (1–10, 11–20, 21–30, 31–40, 41–50%) as within-subjects factors and Group (deaf, hearing) as between-subjects factor. Generally, Greenhouse–Geisser corrected *F* and *p*-values are reported in cases of violations of the sphericity assumption, and *post-hoc t*-tests using a Bonferroni correction were performed where appropriate. If not stated otherwise, a *p*-value of 0.05 or below was deemed statistically significant. The redundancy gain was assessed separately in each group and defined as the difference of the mean of the bounding sum (V + T) and the mean of the visuo-tactile condition (VT) as a proportion of the bounding sum (see Girard et al., 2011). These values were averaged over the five decile bins and then compared between the two groups using an independent-samples two-tailed *t*-test.

#### Electroencephalography data processing

Data processing was performed with MATLAB (MathWorks) software using custom scripts and EEGLAB version 10.2.2.4b (Delorme and Makeig, 2004). The preprocessing was done using a two-step procedure, optimized for artifact correction with independent component analysis (ICA) (e.g., Debener et al., 2010). The first step was performed in order to find ICA weights. For this, the raw data were offline filtered between 1 and 40 Hz. Intervals containing unique, non-stereotyped artifacts were rejected and extended infomax ICA (Bell and Sejnowski, 1995) was applied to the remaining data. In the second step, the resulting ICA weights were applied to raw data filtered with a low pass filter of 40 Hz. The sampling rate was reduced to 250 Hz. These data were then segmented from −200 to 1000 ms relative to stimulus onset. Epochs containing unique, non-stereotyped artifacts were rejected, and artifact activity related to eye blinks, horizontal eye movements, electrocardiographic artifacts, as well as other sources of non-cerebral activity were removed by means of ICA (Jung et al., 2000a,b). Trials for which a response was missing were also excluded from the analysis. Stimulus presentation was lateralized; that is, stimuli were presented either on the left side or on the right. To prevent confounds between contralateral and ipsilateral responses with the lateralized stimulation, for the ERP analysis we took the mirror image of responses to right sided stimulation: Data from right sided stimulation were flipped such that electrodes on the right side changed to the respective position on the left side and vice versa. The midline electrodes stayed at their original position. These mirror imaged data were pooled together with the data from actual left sided stimulation. These pooled data were the basis for all following EEG analyses. Thus, all data were analyzed and illustrated as if they were left sided stimulation. Electrodes on the right side therefore represent contralateral responses.

#### Analysis of ERPs

Brain responses of deaf and hearing participants were compared in the unisensory visual and tactile conditions. Additionally, multisensory interactions were assessed by using the additive model stating that processing of a bisensory stimulus reflects a summation of separate processing mechanisms of the respective unisensory stimuli. A deviation from this model is interpreted as evidence for multisensory interaction between the sensory modalities. Thus, the processing of the bisensory stimulus would be related to the combined presentation of the stimuli rather than to independent processing of the respective unisensory stimuli. The assumptions of the additive model can be expressed in an equation as follows: VT = V + T. However, if there is activity that is inherent to all stimulus conditions, such as anticipatory slow wave potentials or a P300, this approach can lead to artifactual apparent interaction effects. When rearranging the above stated equation to T = VT – V for example, this common activity will be present in T, but will be subtracted out in VT – V, creating an artificial difference between the two sides of the equation (Teder-Salejarvi et al., 2002). In order to avoid artificial interactions due to late common activity as with the P300 (e.g., response related activity), the analysis of multisensory interactions was restricted to early activation, namely the P100 and N200 peaks (for a similar approach, see Besle et al., 2004; Stekelenburg and Vroomen, 2007). Therefore, in the analysis of the P300, only unisensory responses were included and the additive model was not applied. Furthermore, to prevent effects of common anticipatory activity, for each participant, the time locked average of the no stimulus events (nostim) was added to the event-related potentials (ERPs) in the VT condition, resulting in: VT + nostim = V + T (for a similar approach, see e.g., Talsma and Woldorff, 2005; Mishra et al., 2007, 2010; Senkowski et al., 2011).

Including the interaction term (int), the additive model equation becomes VT + nostim = V + T + int, an equation that holds regardless of int. We rearranged the equation as T + int = VT + nostim – V, in order to compare the unisensory tactile response T with the response term [VT + nostim – V] that can thus now be seen to differ from T only by the interaction. This approach seeks to understand multisensory interaction by comparing the unisensory responses with their estimated counterparts in a multisensory context. In the absence of multisensory interaction [VT + nostim – V] would be equal to T. In this case, the simultaneous presentation of a visual stimulus would have had no effect on the processing of the tactile stimulus. If the response term [VT + nostim – V] differed from T, however, we would take this as evidence that the tactile response was modulated by the visual stimulation. Similarly, we also rearranged the model equation as V + int = VT + nostim – T. The logic behind this follows the same principle as described above. If there were multisensory interaction, the response term [VT + nostim – T] would differ from the unisensory visual response V. Although this method looks separately at each sensory modality, it remains likely however that the interaction term contains at least some proportion of visual and tactile as well as other non-specified activity.

**Figure 1:**
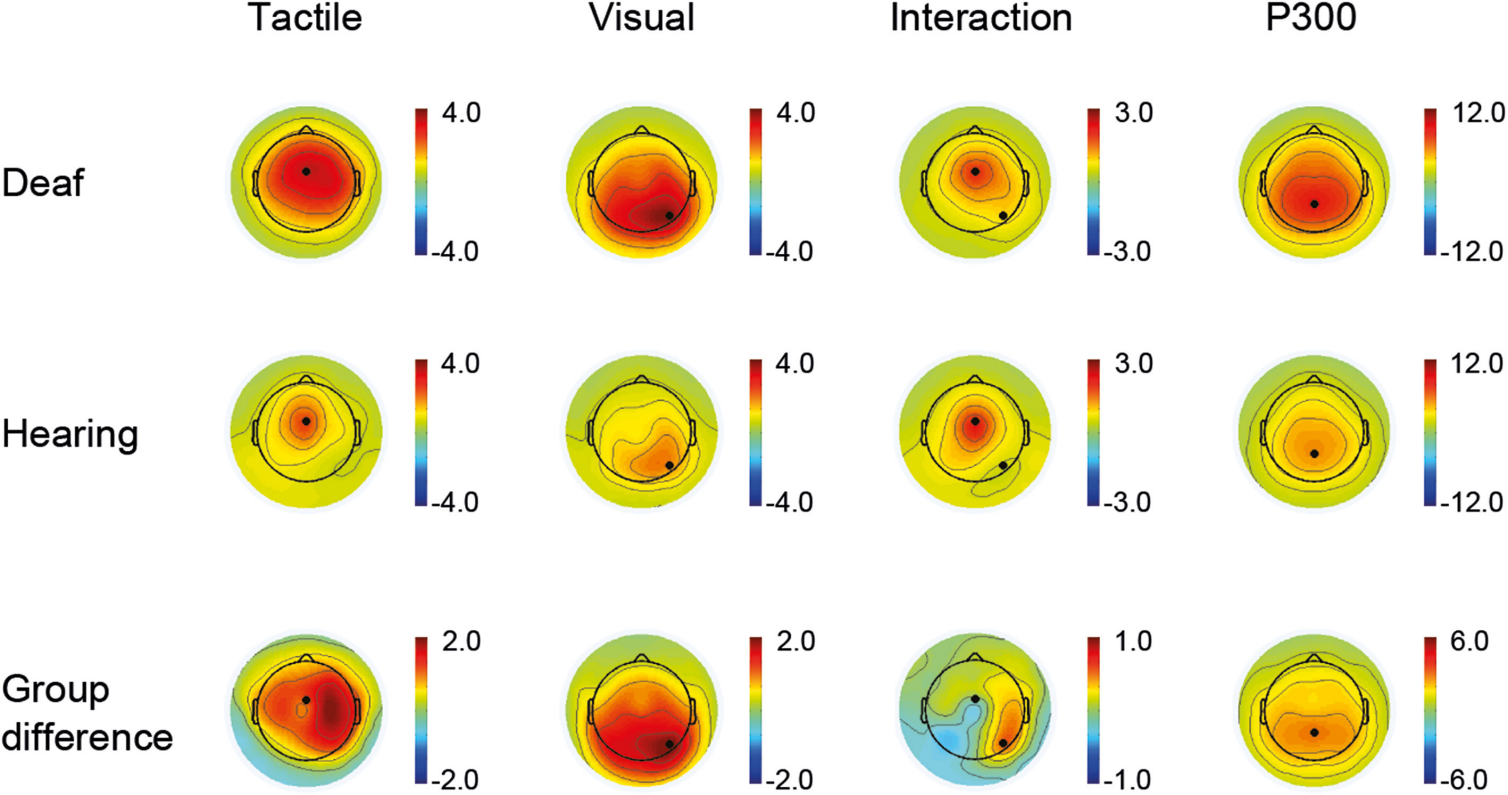
**Active channels**. Channel activity is defined as the standard deviation across time of the grand average data for each group separately and for group differences. The time window for the tactile and visual condition as well as the interaction (VT + nostim − V − T) was 80–300 ms (first three columns). A clear single peak channel is evident in both groups for each sensory modality. For the P300, shown as an average of the visual and tactile conditions, a time window of 200–500 ms was used (fourth column).

Time windows for the ERP peak detection were defined as follows: P100: 80–140 ms, N200: 152–252 ms, and P300: 260–560 ms. Electrode sites were determined by visual inspection of the channel activity plots shown in Figure 1. Channel activity is defined as the standard deviation across time (80–300 ms) of the grand average data for each group separately. A clear single peak channel is evident in both groups for each sensory modality, and these channels were therefore selected as best representing the respective modality in all subsequent analysis. Thus, the unisensory tactile response T and the [VT + nostim – V] response (P100, N200) were derived at a fronto-central electrode site, and the unisensory visual response V and the [VT + nostim – T] response (P100, N200) were derived at a right occipital electrode site. P300 peaks were obtained similarly from a parietal electrode site (see Figure 1). Semi-automatic peak detection was performed on single-subject averages for each condition: V, T, [VT + nostim – T], [VT + nostim – V] (the latter two only for P100 and N200). Peak latencies for each component were defined as the most positive or negative peak occurring in the corresponding time window after stimulus onset. Similarly, peak amplitudes for each component were measured as the average amplitude across an interval containing the local maximum or minimum value plus/minus one sample (i.e., 8 ms). Analysis of earlier peaks revealed no group differences and is not reported.

A 2 × 2 mixed ANOVA with Stimulus type (V, [VT + nostim – T]) as within-subjects factor and Group (deaf, hearing) as between-subjects factor was conducted on P100 and N200 amplitudes and latencies. Another Stimulus type (T, [VT + nostim – V]) × Group (deaf, hearing) mixed ANOVA was computed for P100 and N200 amplitudes and latencies. A 2 × 2 mixed ANOVA was conducted for unisensory P300 amplitudes and latencies with Modality (V, T) as within-subjects factor and Group (deaf, hearing) as between-subjects factor.

## Results

### Behavioral results

The mean detection rate was 98.9% for the visual (V), 98.0% for the tactile (T) and 99.5% for the redundant signals (VT) condition with no significant differences between the two groups. An overview of the mean reaction times (RTs) in the two groups is shown in Table 2. In all conditions, mean RTs were between 300 and 400 ms. The Stimulus condition (V, T, VT) × Group (deaf, hearing) ANOVA on RTs revealed a main effect of Stimulus condition, *F*_(1.3, 22.9)_ = 63.57, *p* < 0.001, but no main effect of Group, *F*_(1, 18)_ = 0.48, *p* = 0.498. The interaction of Stimulus condition by Group was significant, *F*_(1.3, 22.9)_ = 4.87 *p* = 0.030. To assess differences in unisensory RTs between deaf and hearing participants, independent-samples two-tailed *t*-tests were conducted separately for the visual and the tactile condition, however, without significant results. To test for the redundant signals effect, subsequent paired-samples two-tailed *t*-tests were conducted for each group separately. For deaf participants, mean RTs to redundant signals (*M* = 329 ms) were faster than those to tactile (*M* = 366 ms), *t*_(9)_ = 4.93, *p* = 0.006 and visual stimuli (*M* = 391 ms), *t*_(9)_ = 11.16, *p* < 0.001. Thus, the deaf group showed a redundant signals effect. Similarly, hearing participants responded on average faster to redundant signals (*M* = 309 ms) than to tactile (*M* = 367 ms), *t*_(9)_ = 8.62, *p* < 0.001, and to visual stimuli (*M* = 358 ms), *t*_(9)_ = 12.79, *p* < 0.001. Hence, the hearing also showed a redundant signals effect. Furthermore, paired-samples two-tailed *t*-tests revealed no significant difference between RTs to tactile and visual stimuli, either in the deaf, *t*_(9)_ = 2.27, *p* = 0.395, or in the hearing group, *t*_(9)_ = 0.95, *p* = 1.

**Table 2:** **Mean response times ± standard deviation**.

	**Deaf**	**Hearing**
V	391 ± 70	358 ± 32
T	366 ± 82	367 ± 39
VT	329 ± 72	309 ± 28

**Figure 2:**
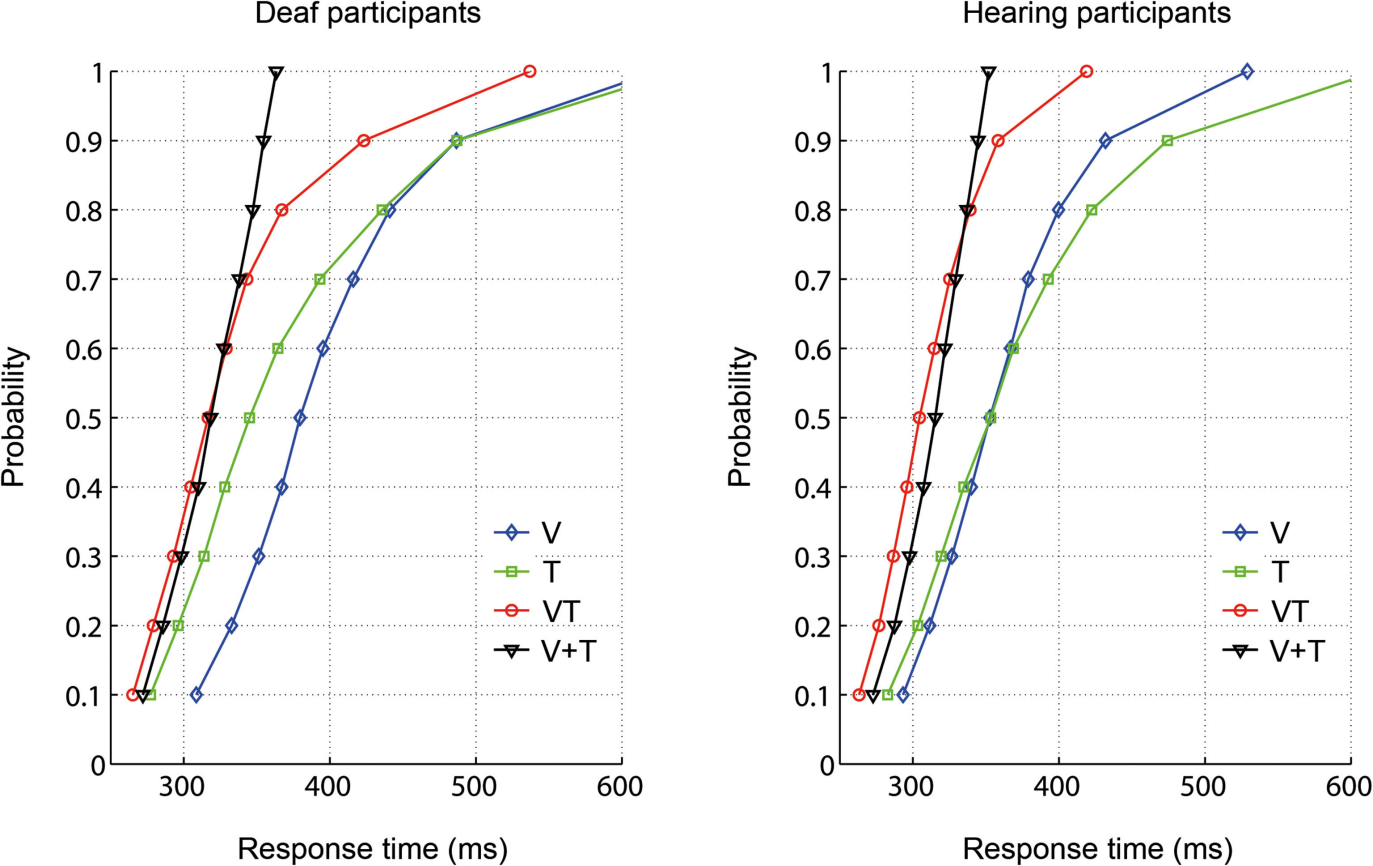
**Response time distribution for visual (V), tactile (T), and redundant signals (VT) as well as the bounding sum (V + T)**.

To examine whether the RTs for bisensory stimuli exceeded the facilitation predicted by a race model, the race model inequality was tested (Miller, 1982). Any violation against the race model inequality indicates that stimuli are not processed in separate channels, that is, there is evidence of an underlying coactivation mechanism. On an individual level, descriptively, all participants except for one deaf, showed violations of the race model. The cumulative density functions (CDFs) for each group are depicted in Figure 2. In each group, the redundant signals condition (VT) and the bounding sum (V + T) were compared for the five fastest deciles (10-percentile bins: 0–50%) using a one-tailed *t*-test with Bonferroni correction to adjust for multiple comparisons. The race model was violated in both groups. In particular, there were significant race model violations in all five tested decile bins for the hearing group whereas the deaf group showed a violation only in the first bin (see Table 3).

**Table 3:** **Comparison of redundant signals and bounding sum in each decile**.

	**Deaf**			**Hearing**		
**Decile**	**VT**	**V + T**	***p***	**VT**	**V + T**	***p***
0.10	265	272	0.037*	263	273	0.012*
0.20	279	286	0.095	277	287	0.001*
0.30	293	298	0.312	287	298	0.003*
0.40	305	310	0.130	296	307	0.001*
0.50	317	318	1	305	315	0.001*

*VT corresponds to the redundant signals condition and V + T to the bounding sum. Paired-samples one-tailed t-tests with Bonferroni correction for multiple comparisons were conducted in each group. An asterisk indicates significance (p < 0.05)*.

The Stimulus type (VT, V + T) × Decile (1–10, 11–20, 21–30, 31–40, 41–50%) × Group (deaf, hearing) ANOVA on CDFs revealed a main effect of Stimulus type, *F*_(1, 18)_ = 38.43, *p* < 0.001, with faster responses for redundant signals (*M* = 289 ms) than for the bounding sum (*M* = 297 ms). As expected, there was also a main effect of Decile, *F*_(1.1, 20.5)_ = 148.38, *p* < 0.001, with faster responses in lower decile bins. Moreover, the interaction between Stimulus type and Group was significant, *F*_(1, 18)_ = 4.62, *p* = 0.046. Subsequent paired-samples one-tailed *t*-tests for each group showed that responses were significantly faster for redundant signals (VT) than for the bounding sum (V + T) in both groups, deaf [*t*_(9)_ = 3.17, *p* = 0.023], and hearing [*t*_(9)_ = 5.43, *p* < 0.001]. An independent-samples two-tailed *t*-test comparing the redundancy gain between deaf and hearing participants was significant, *t*_(18)_ = 2.33, *p* = 0.032, with higher values in the hearing than in the deaf group (*M* = 3.5 vs. 1.7%). That is, hearing controls benefited twice as much from redundant stimulation than did deaf participants.

### Electroencephalography results

#### ERPs: responses at a fronto-central site (P100, N200)

**Figure 3:**
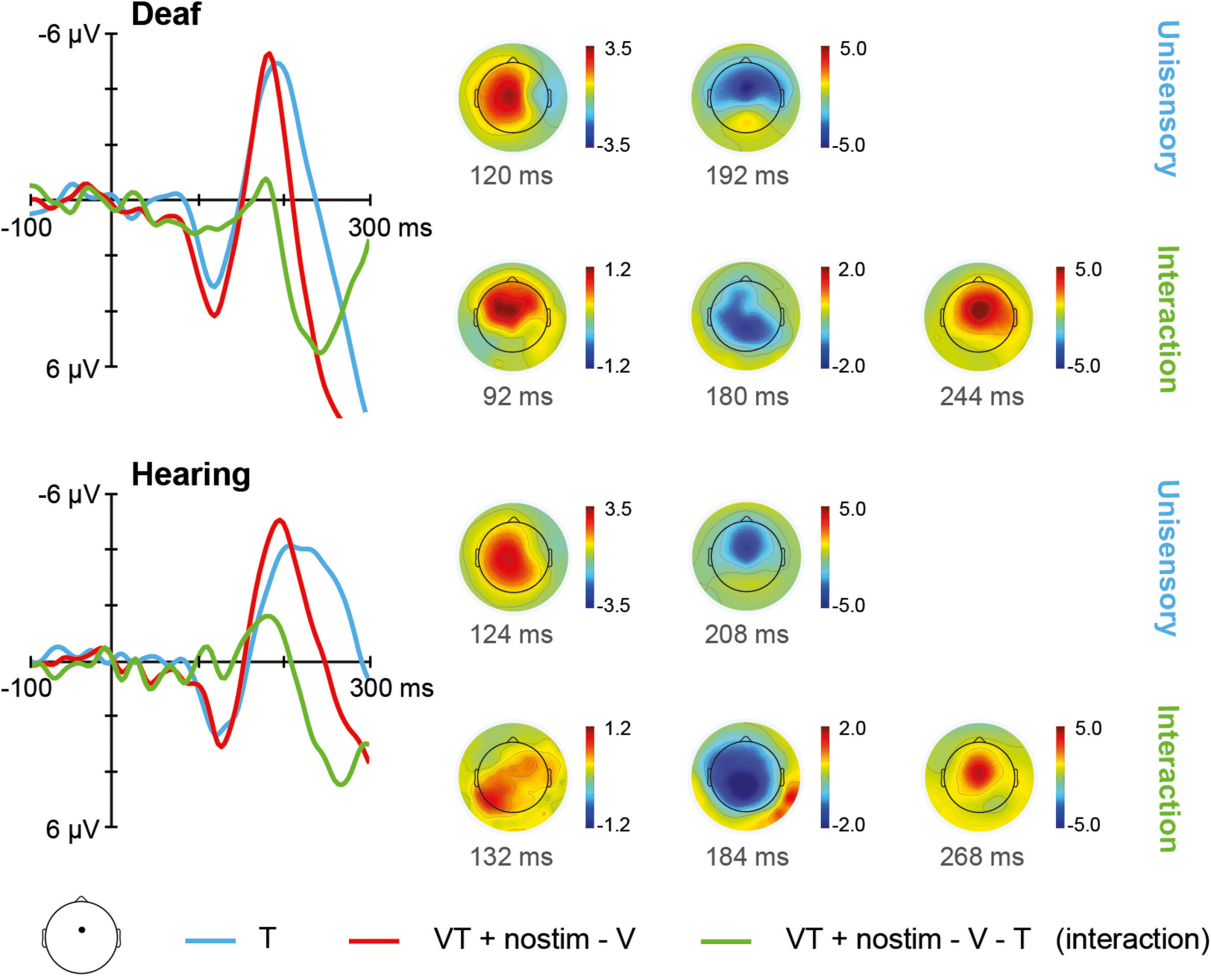
**Tactile stimulation**. **Left**: Grand average ERPs recorded at a fronto-central electrode site are shown for deaf and hearing participants. The unisensory tactile response T is depicted in blue whereas the [VT + nostim – V] response is depicted in red. The [VT + nostim – V – T] response reflecting the interaction is illustrated in green. N200 latencies to unisensory tactile stimuli were shorter in the deaf than the hearing group. Moreover, shorter latencies for [VT + nostim – V] than unisensory tactile T responses (i.e., multisensory enhancement) were observed in hearing, but not in deaf participants for the N200 peak. **Right**: Topographies of grand average ERPs are shown for deaf and hearing participants. The topographies at the P100 and N200 peaks are given separately for the unisensory T response. Topographies of the [VT + nostim – V – T] response (i.e., the interaction) are also shown.

The ERPs for unisensory tactile T and [VT + nostim – V] responses in deaf and hearing participants are depicted in Figure 3. For unisensory tactile stimuli, the grand average at the fronto-central electrode showed a positive peak around 120 ms (referred to as P100) and a negative peak around 200 ms (referred to as N200). These peaks were verified by visual inspection of the global field power (GFP) data.

The Stimulus type (T, [VT + nostim – V]) × Group (deaf, hearing) ANOVA on P100 amplitudes revealed a marginally significant main effect of Stimulus type, *F*_(1, 18)_ = 3.50, *p* = 0.078, with larger amplitudes for [VT + nostim – V] than for unisensory tactile responses T (*M* = 4.0 vs. 3.2 μV), indicating a tendency for multisensory interactions about 100 ms after stimulus presentation in both deaf and hearing participants. For P100 latencies, the main effect of Stimulus type also approached significance, *F*_(1, 18)_ = 3.45, *p* = 0.080, due to an earlier peak of the [VT + nostim – V] response as compared to the unisensory tactile response T (*M* = 122 vs. 126 ms).

The ANOVA on N200 latencies showed a main effect of Stimulus type, *F*_(1, 18)_ = 18.68, *p* < 0.001, and Group, *F*_(1, 18)_ = 11.67, *p* = 0.003. The interaction was also significant, *F*_(1, 18)_ = 7.06, *p* = 0.016. Subsequent paired-samples two-tailed *t*-tests were conducted for each group separately. A multisensory enhancement, with shorter latencies for [VT + nostim – V] than for unisensory tactile responses T, was observed in hearing (*M* = 195 vs. 230 ms), *t*_(9)_ = 3.96, *p* = 0.010, but not in deaf participants, *t*_(9)_ = 1.77, *p* = 0.332, indicating group differences in multisensory interactions. In order to assess group differences in unisensory processing, an independent-samples two-tailed *t-test* comparing the latencies of deaf and hearing participants separately for unisensory tactile responses was conducted. A significant group difference was found, *t*_(18)_ = 3.46, *p* = 0.008, with shorter N200 latencies for deaf than for hearing participants (*M* = 192 vs. 230 ms).

#### ERPs: responses at an occipital site (P100, N200)

**Figure 4:**
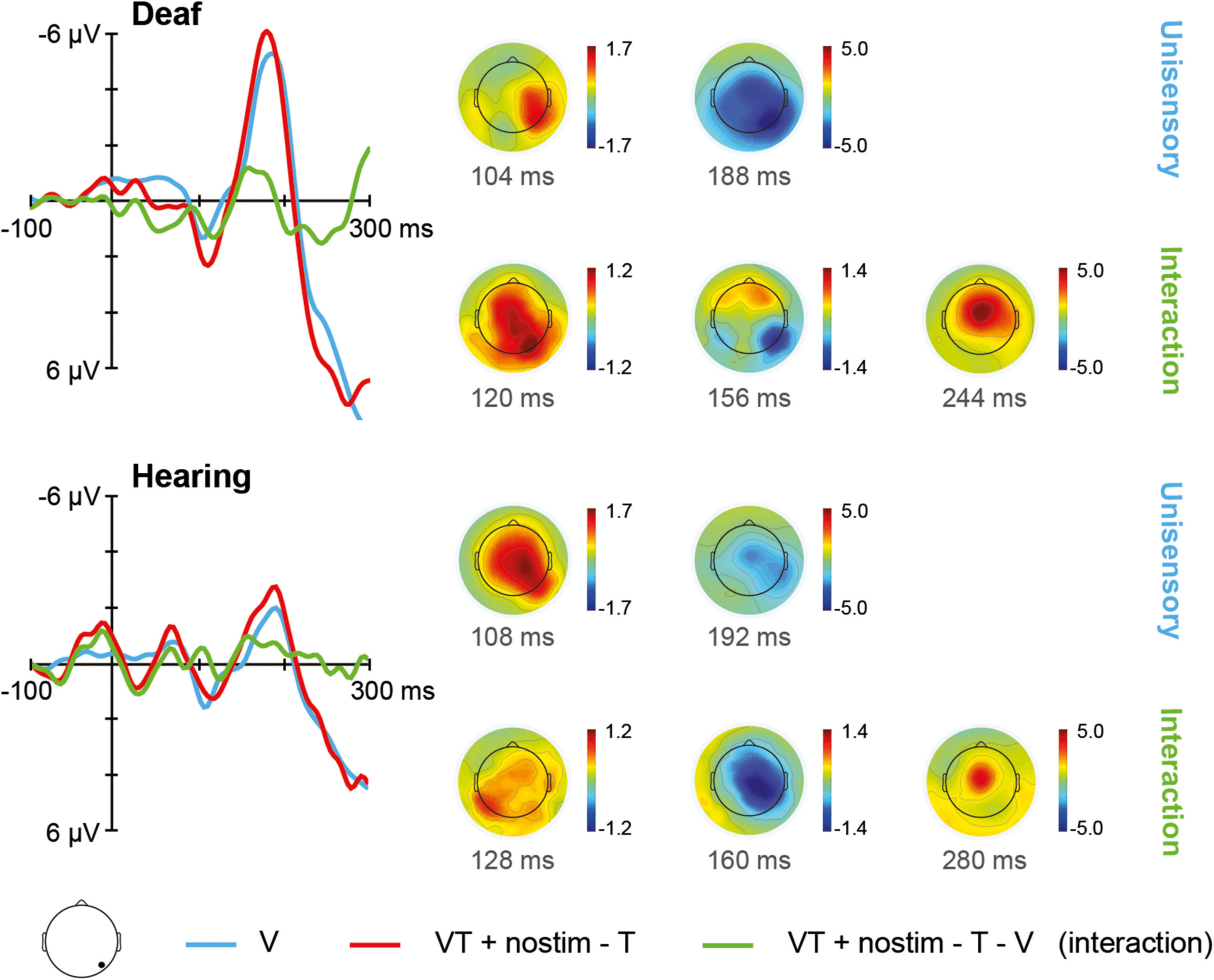
**Visual stimulation**. **Left**: Grand average ERPs recorded at an occipital electrode site are shown for deaf and hearing participants. The unisensory visual response V is depicted in blue whereas the [VT + nostim – T] response is depicted in red. The [VT + nostim – T – V] response reflecting the interaction is illustrated in green. N200 amplitudes were stronger in the deaf than in the hearing group. Moreover, stronger N200 amplitudes and shorter N200 latencies for [VT + nostim – T] than for unisensory visual V responses (i.e., multisensory enhancement) were observed in both groups. **Right**: Topographies of grand average ERPs are presented for deaf and hearing participants. The topographies at the P100 and N200 peaks are given separately for the unisensory V and [VT + nostim − T] response. Topographies of the [VT + nostim – T – V] response (i.e., the interaction) are also shown.

The ERPs for unisensory visual V and [VT + nostim – T] responses in deaf and hearing participants are depicted in Figure 4. For unisensory visual stimuli, the grand average at the occipital electrode showed a positive peak around 108 ms (referred to as P100) and a negative peak around 188 ms (referred to as N200). These peaks were verified by visual inspection of the GFP data.

The Stimulus type (V, [VT + nostim – T]) × Group (deaf, hearing) ANOVA on N200 amplitudes revealed a main effect of Stimulus type, *F*_(1, 18)_ = 6.05, *p* = 0.024, with stronger amplitudes for the [VT + nostim – T] than for the unisensory visual V response (*M* = −5.4 vs. −4.4 μV). Additionally, the main effect of Group was significant, *F*_(1, 18)_ = 4.66, *p* = 0.045, with deaf participants showing stronger amplitudes than their hearing counterparts (*M* = −6.4 vs. −3.5 μV). For N200 latencies, there was a main effect of Stimulus type, *F*_(1, 18)_ = 6.53, *p* = 0.020, with shorter latencies for [VT + nostim – T] than for unisensory visual responses V (*M* = 179 vs. 185 ms). No other effects or interactions reached statistical significance.

#### ERPs: post-hoc interaction analysis

Active channels with respect to visuo-tactile interactions are shown in Figure 1 (third column). In both groups the distribution of activity closely resembled that of the unisensory tactile responses. Additionally, the largest group difference in this interaction activity occurred at the right occipital location identified as most active following visual stimulation. Together, this pattern indicates, firstly, that there is a general modulation of tactile responses with bisensory stimulation in both groups, and, secondly, that although any modulation of visual activity with bisensory stimulation is weak, it is nevertheless stronger in the deaf group.

To test these indications statistically, we performed a *post-hoc* analysis of peak amplitudes in the interaction waveforms. Based on grand averages, at the fronto-central site, three peaks were identified at around 128 ms, 180 ms, and 256 ms, respectively. One-sample *t*-tests on interaction amplitudes revealed significant deviations from zero at all three peaks, peak 1: *t*_(19)_ = 2.18, *p* = 0.042; peak 2: *t*_(19)_ = 2.06, *p* = 0.054; peak 3: *t*_(19)_ = 5.19, *p* < 0.001); in each case absolute amplitude was larger for [VT + nostim – V] than for T (0.78 μV; 1.19; 4.42). Thus, a modulation of tactile responses with bisensory stimulation was confirmed. Independent-samples *t*-tests showed no significant group differences in interactions at any peak.

At the right occipital site, three peaks were identified at around 124 ms, 160 ms, and 252 ms, respectively. There was a marginally significant deviation at peak 1, *t*_(19)_ = 0.85, *p* = 0.072, and a significant deviation from zero for peak 2, *t*_(19)_ = 2.10, *p* = 0.049, with absolute amplitudes being larger for [VT + nostim – T] than for V (0.85 μV; 1.07), and no significant difference at peak 3, confirming the general weakness of visual interactions. There were no significant group differences in interactions at any peak, so we could not confirm indications of stronger visual interactions in the deaf group.

#### P300 group comparisons

**Figure 5:**
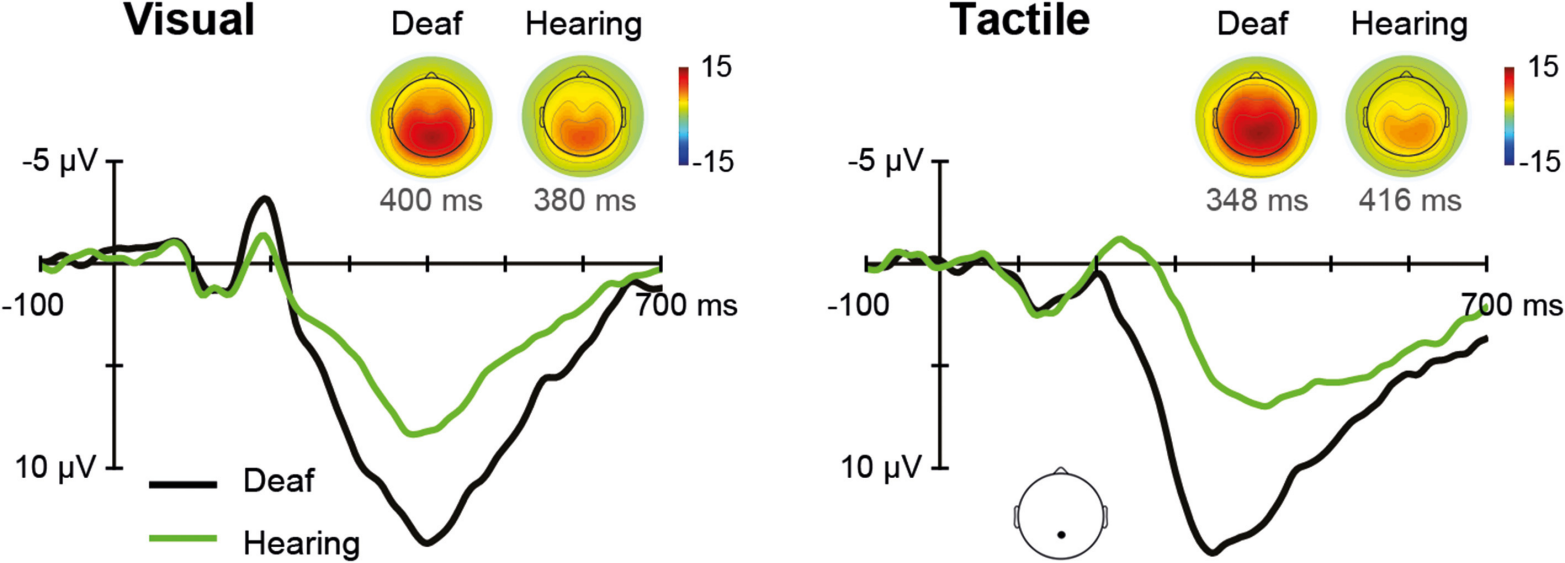
**Unisensory ERPs**. Grand average ERPs recorded at a parietal electrode site are illustrated for deaf and hearing participants. The left panel shows unisensory visual and the right panel unisensory tactile responses. P300 topographies of grand average ERPs are shown separately for deaf and hearing participants.

The ERPs for each sensory modality, separately for deaf, and hearing participants, are depicted in Figure 5. In the unisensory conditions, the grand average for the parietal electrode showed a positive peak around 396 ms for both visual and tactile responses (referred to as P300).

The Modality (V, T) × Group (deaf, hearing) ANOVA on P300 amplitudes revealed a main effect of Group, *F*_(1, 18)_ = 8.08, *p* = 0.011, with larger amplitudes for deaf than for hearing participants (*M* = 15.0 vs. 9.0 μV), indicating a more pronounced P300 for both visual and tactile stimuli in the deaf. Additionally, the Modality by Group interaction was significant, *F*_(1, 18)_ = 4.52, *p* = 0.048. Follow-up independent-samples *t*-tests indicated a significant group difference for tactile stimulation (15.8 vs. 8.7), *t*_(18)_ = 3.15, *p* = 0.012, whereas the group difference for visual stimulation was marginally significant (14.2 vs. 9.3), *t*_(18)_ = 2.34, *p* = 0.062. For P300 latencies, there were no significant effects.

## Discussion

The present study investigated unisensory and multisensory processing with auditory deprivation. Visual, tactile, and a combination of both stimuli were presented to congenitally deaf and hearing individuals in a speeded detection task. Regarding unisensory responses, we found earlier tactile and more pronounced visual and tactile neural responses for the deaf than for hearing controls. These group differences in the ERPs were not reflected in the behavioral results. In general, visuo-tactile interactions were present for deaf and hearing participants, but with different characteristics.

### Unisensory visual and tactile stimulation

In the present study, the ability to detect simple visual stimuli was not superior in the deaf as indicated by the lack of a group difference in RTs to either unisensory visual or tactile stimuli. This was surprising given that previous studies using a similar detection task have reported faster responses to visual stimuli in deaf than in hearing individuals (e.g., Bottari et al., 2010; Heimler and Pavani, 2014). Similarly, the ability to detect simple tactile stimuli was not enhanced in deaf over hearing participants as was recently shown by Heimler and Pavani (2014).

Regarding the EEG, for the *visual* stimuli, deaf participants showed more pronounced N200 amplitudes than hearing controls. This is consistent with Neville et al. (1983) and Hauthal et al. (2014) who also reported greater neural responses in deaf compared to hearing individuals. Similarly, Bottari et al. (2011) found, at least descriptively, a larger positivity around 100–150 ms in deaf participants when they detected peri-foveal and peripheral visual stimuli. Together with the present findings, this suggests alterations in visual processing with deafness. Regarding *tactile* stimuli, we found shorter N200 latencies in the deaf compared to hearing participants. Although the earlier N200 latencies were not reflected in faster RTs in our deaf participants, they suggest temporally more efficient neural processing compared to hearing individuals. Moreover, earlier visual N1 latencies have been found to be related to lower processing effort in visual tasks (Callaway and Halliday, 1982; Fort et al., 2005). Thus, the earlier N200 latencies in deaf participants could point to a reduced processing effort for tactile stimuli in this group, also suggesting a more efficient processing of tactile stimuli in the deaf. We found tactile P300 amplitudes to be significantly larger in deaf than in hearing participants whereas the group difference for visual P300 amplitudes pointed into the same direction approaching significance. P300 amplitudes reflect stimulus classification and evaluation (for a review, see Polich, 2007), this suggests group differences regarding higher-level cognitive functions.

In general, the differences in processing of visual and tactile targets between deaf and hearing participants were not related to behavioral advantages for the deaf in stimulus detection as has been reported previously by Bottari et al. (2011) for visual stimulation. Thus, regarding *unisensory* visual and tactile stimulation, we did not find a relationship between cross-modal reorganization and behavioral enhancements. Nevertheless, the earlier neural responses to unisensory tactile stimulation in deaf compared to hearing participants point to a more efficient neural processing with auditory deprivation.

### Multisensory interactions

The present study examined behavioral and electrophysiological correlates of visuo-tactile processing in deaf and hearing individuals. Both participant groups showed a redundant signals effect. RTs were faster, on average, when a visual and a tactile stimulus were presented together, compared to when each was presented alone as has been previously shown for hearing individuals (e.g., Hecht et al., 2008; Girard et al., 2011). The race model inequality (Miller, 1982) was violated in deaf and hearing participants suggesting a coactivation mechanism in both groups. Accordingly, RTs to the redundant signals were too fast to be explained by probability summation. Deaf and hearing participants, however, differed in how much they benefited from redundant signals. The redundancy gain was, on average, about twice as large in hearing compared to deaf participants. This result contradicts the finding of Karns et al. (2012), who reported deaf but not hearing participants to be susceptible to a visuo-tactile version of the Shams' illusion (Shams et al., 2005). A look at response time differences between sensory modalities in the deaf group suggests a possible alternative explanation for the reduced redundancy gain found for the deaf in the present study. Numerically, deaf participants responded more slowly to visual than to tactile stimuli, a difference not seen in their hearing counterparts. If this difference reflects a relative delay in low-level visual compared with tactile processing, and if this delay persists even with concurrent stimulation, then the potential for multisensory interaction is reduced because of the reduced temporal overlap of early unisensory processes. The response time difference between the visual and tactile condition in deaf individuals, however, did not reach statistical significance and is therefore rather unlikely to be the reason for the reduced redundancy gain observed in the deaf.

Research in blind individuals also points to a reduction of interplay between the remaining sensory modalities with visual deprivation (Hötting and Röder, 2004; Champoux et al., 2011; Occelli et al., 2012; but see Collignon et al., 2009, uncrossed hand posture). Hötting and Röder (2004), for example, tested congenitally blind and sighted participants in an audio-tactile version of the Shams' illusion and found the illusion to be less pronounced in blind participants. Collignon et al. (2009), however, showed violations of the race model, indicating a coactivation mechanism, in an audio-tactile localization task in early blind, late blind, and sighted participants, when hands were uncrossed. Regarding auditory deprivation, temporarily deaf individuals whose hearing had been restored by a CI have been tested with the same kind of audio-tactile illusion that had been used previously by Hötting and Röder (2004) in blind individuals (Landry et al., 2013). This illusion was perceived in normally hearing participants, but not in CI users, which is consistent with the present finding of reduced redundancy gain in congenitally deaf individuals. Thus, behavioral findings in blind individuals point to similar (Collignon et al., 2009) or reduced (Hötting and Röder, 2004; Champoux et al., 2011; Occelli et al., 2012) audio-tactile interaction compared with sighted controls, whereas findings for deaf individuals and CI users (who have experienced a period of auditory deprivation) seem to be contradictory. On the one hand, previous results suggest more pronounced visuo-tactile interaction in deaf individuals (Karns et al., 2012). On the other hand, they point to a reduction in visuo-tactile interaction in deaf individuals (present study), as well as in audio-tactile and audio-visual interaction in late-implanted CI users (Gilley et al., 2010; Landry et al., 2013). Note that even very proficient CI users have a hearing impression that is different from that of normal hearing individuals. Therefore, in CI users, general multisensory integration abilities can only be assessed by presenting stimuli that can be perceived without any restrictions, that is, visual and tactile. Since the aim of implanting a CI is a hearing impression that is as close to that of normal hearing individuals as possible, including full associated functionality such as multisensory integration, studies testing audio-visual and audio-tactile interactions are needed. Results of such studies should, however, always be interpreted in conjunction with the CI users' general ability to integrate multisensory information as best reflected by visuo-tactile interactions both before and after implantation.

In the present study, multisensory interactions in *ERPs* were examined by applying the additive model. In particular, we estimated the effects on each single sensory system and compared these between deaf and hearing participants. To assess the influence of touch on vision, we compared the unisensory visual V with the [VT + nostim – T] response. Conversely, the influence of vision on touch was assessed by comparing the unisensory tactile T with the [VT + nostim – V] response. Thus, if these responses at a particular electrode site were the same, there would be no evidence of multisensory interaction. In the cases where they did differ, on the other hand, we took this as evidence that the simultaneous presentation of a stimulus of one sensory modality had an influence on the processing of the stimulus of the respective other modality. This would be interpreted as evidence for multisensory interaction. Although our method looks separately at each sensory modality, the possibility of a contribution from other sources nevertheless remains.

In general there was a much stronger modulation of tactile than of visual responses with bisensory stimulation. There was a tendency for tactile responses being modulated as early as the P100 in both deaf and hearing participants. In particular the [VT + nostim – T] response was more pronounced and peaked earlier than the unisensory visual response. Similarly, visual N200 responses were earlier for the [VT + nostim − T] as compared to unisensory visual stimulation. Thus, visuo-tactile interactions occurred in both deaf and hearing participants at both the P100 and the N200. This was also reflected in behavior with both groups showing a redundant signals effect due to a coactivation mechanism, albeit to a different extent. Similarly, these results are consistent with earlier reports of multisensory interactions with visual and tactile stimuli (e.g., Schürmann et al., 2002). Nevertheless, an important group difference was evident in our data, in that the multisensory enhancement, in terms of shorter N200 latencies with bisensory than with unisensory stimulation, was seen only in the hearing participants, and not in the deaf. Thus, hearing but not deaf participants' *tactile* responses were modulated by the simultaneous presentation of a *visual* stimulus. This result also corresponds with the smaller behavioral redundancy gain we found in our deaf than in our hearing participants.

Evidence of modulation of visual responses with bisensory stimulation was weaker than for tactile. Indeed, there were no modulations of the visual P100 in either group. At the N200 peak, there was again evidence of shorter latencies with bisensory stimulation, but the 6 ms reduction was smaller than the 22 ms seen for tactile responses. The one effect evident with visual but not with tactile responses was the greater N200 amplitude with bisensory than with unisensory stimulation. In terms of group differences there were some indications of greater modulation of visual responses in deaf compared with hearing participants by simultaneous tactile stimulation, as our visual right-occipital site was more active during interactions in the deaf group than in the hearing. This would have been in direct contrast to the visual modulation of tactile responses in the hearing. However, the effect could not be statistically confirmed.

We limited our interaction analysis to two scalp sites identified as best representing tactile and visual activity, respectively. Indeed analysis of interactions indicated that activity was strongest generally at the putative tactile site, and that group differences in activity were strongest at the putative visual site. Thus, we focused only on modulations of unisensory activity. Recent data however suggests that cross-modal stimuli may engage distinct configurations of source generators in addition to just increasing or reducing the strength of a response (Vidal et al., 2008). Our sensor data do not allow us to make any determination in this regard, and our experimental design precludes a similar analysis. Nevertheless future studies may benefit from taking such considerations into account where applicable.

The reduced multisensory interaction in behavior and partly in the ERPs of the deaf cannot be explained by the perceptual deficiency hypothesis, which states that deaf individuals perform at a lower level compared to hearing controls. This hypothesis suggests that a contribution of all sensory systems would have been necessary to develop multisensory integration abilities. The present findings of multisensory interaction at the N200 and as a tendency at the P100 in both groups, however, make this explanation very unlikely. The visuo-tactile results of the present study could potentially be explained by inverse effectiveness, the phenomenon by which multisensory stimuli are more effectively integrated when the unisensory responses are relatively weak (Stein and Meredith, 1993; see also Diederich and Colonius, 2004; Rach et al., 2011; Senkowski et al., 2011). A possible explanation is that, because intense stimuli are more reliable (than less intense stimuli) information from another sensory modality is not needed, resulting in reduced multisensory integration (see e.g., Ernst and Bülthoff, 2004). In the present study, the N200 latency in response to unisensory tactile stimulation was shorter (and therefore eventually more reliable) in deaf than hearing participants which could have led to less multisensory interaction in the deaf. Indeed, a multisensory enhancement with somatosensory evoked potential N200 latencies being modulated by visual stimulation was observed not in deaf, but in hearing participants, for which the unsisensory tactile input may have been less reliable as compared to the deaf (for similar considerations regarding the blind, see Hötting et al., 2004).

Interestingly, multisensory processing has also been reported to be reduced in (hearing) young compared to (hearing) older individuals (Laurienti et al., 2006; Peiffer et al., 2007; Diederich et al., 2008; Mahoney et al., 2011). For example, Mahoney et al. (2011) reported a lower degree of visuo-tactile integration in younger than in older adults. Moreover, these multisensory effects occurred irrespective of spatial location for both stimuli presented within the same and across different hemifields (Mahoney et al., 2014). The underlying reason for these observations is not yet clear. One possible explanation in line with the principle of inverse effectiveness is that with enhanced multisensory integration, older individuals can compensate for deficits they show in unisensory processing (for a review, see Mozolic et al., 2011). This example demonstrates that a reduction in multisensory interaction does not necessarily reflect a disadvantage, but in the present study might instead have occurred as a consequence of enhanced unisensory processing in the deaf.

Regarding the ERPs, deaf participants also showed more pronounced N200 amplitudes in response to unisensory visual stimulation, as discussed above. However, the N200 latencies of visual ERPs were modulated by simultaneous tactile stimulation in both deaf and hearing participants. Following the rule of inverse effectiveness, regarding the more pronounced visual neural responses in the deaf as compared to hearing controls, one would expect smaller multisensory interactions in the deaf. Therefore, the multisensory results of the present study do not support inverse effectiveness as a general explanation for all of the present results.

Investigating multisensory processing in congenitally deaf individuals helps to shed light on the extent to which the contribution of all sensory systems during maturation is necessary for the development of the ability to integrate information from different sensory systems. The results of the present study suggest that auditory deprivation does not prevent a general ability to integrate multisensory information, but that it has an influence on the extent to which individuals benefit from bimodal stimulation. To further examine this, we need studies that test visuo-tactile interactions in individuals that were deafened later in life (who, up to a certain point in time, went through the same development as normal hearing individuals) and in individuals that show a moderate hearing loss. Are these individuals more similar to the congenitally deaf or to the normal hearing, or are they somewhere between the two? This gives information about whether the development of the ability to integrate multisensory inputs follows an “all-or-nothing” principle or whether it is more of a graduated process.

## Summary

In the present study we found that unisensory visual and tactile stimulus processing was different in the congenitally deaf compared to hearing controls. Firstly, the deaf participants showed larger N200 amplitudes in visual ERPs and shorter N200 latencies in somatosensory ERPs compared to hearing controls. Furthermore, P300 amplitudes were larger in the deaf. This group difference was significant for tactile and approached significance for visual stimulation. Secondly, regarding multisensory processing, both groups showed a redundant signals effect that was attributable to a coactivation of visual and tactile processing. The redundancy gain, however, was less in deaf compared to hearing participants. Multisensory interactions were observed in ERPs at latencies around the N200 peaks and as a tendency for the tactile P100 in both participant groups. In accordance with the behavioral results, an apparent visual modulation of tactile responses was present at N200 latencies in hearing, but not deaf participants. Thus, the ERP findings are consistent with the reduced behavioral redundancy gain in the deaf. Multisensory enhancements could not be explained by perceptual deficiency, but could at least partly be attributed to the principle of inverse effectiveness.

### Conflict of interest statement

The authors declare that the research was conducted in the absence of any commercial or financial relationships that could be construed as a potential conflict of interest.
